# Comparison of static and dynamic balance measurements among chronic and episodic migraine patients

**DOI:** 10.1590/0004-282X-ANP-2020-0319

**Published:** 2021-05-01

**Authors:** Seyhan Dumanlidağ, Aysel Milanlioğlu

**Affiliations:** 1 Yüzüncü Yıl University Faculty of Medicine Department of Neurology Van Turkey Yüzüncü Yıl University, Faculty of Medicine, Department of Neurology, Van, Turkey.

**Keywords:** Migraine Headaches, Posture Balance, Postural Control, Transtornos de Enxaqueca, Equilíbrio Postural, Controle da Postura

## Abstract

**Background:**

Migraine is one of the most frequent and incapacitating headaches, with a high degree of impairment of balance control and postural stability.

**Objective:**

To investigate the effects of episodic and chronic migraine on postural balance through using static and dynamic balance tests.

**Methods:**

The study included 32 chronic and 36 episodic migraine patients and a control group of 36 healthy volunteers. Right/left single-leg static and dynamic balance tests were performed in each group with eyes open and closed using a posturographic balance platform (Techno-body Prokin).

**Results:**

No significant difference was found among episodic and chronic migraine patients and control subjects with regard to eyes-open and eyes-closed area values (eyes-open area values: p=0.559, p=0.414 and p=0.906; eyes-closed area values: p=0.740, p=0.241 and p=0.093, respectively). However, the area values were significantly higher in episodic and chronic migraine patients than in the control group, which indicates that migraine patients may have lower balance performance. Perimeter values were relatively higher which supports the idea that migraine patients have lower balance performance. Additionally, the average number of laps was significantly lower among migraine patients than in the control group, which also implies that migraine patients may have lower balance performance.

**Conclusion:**

Although no significant difference was detected between chronic and episodic migraine patients and the control group and between chronic and episodic migraine patients with regard to balance performance, chronic migraine patients seemed to have relatively lower performance than episodic migraine patients. Further studies with larger numbers of patients are needed, to investigate the relationship between these parameters and balance.

## RESUMO

**Introdução:**

A enxaqueca é uma das dores de cabeça mais frequentes e incapacitantes, com alto grau de comprometimento do controle do equilíbrio e estabilidade postural.

**Objetivo:**

Investigar os efeitos da enxaqueca episódica e crônica no equilíbrio postural por meio de testes de equilíbrio estático e dinâmico.

**Métodos:**

O estudo incluiu 32 pacientes com enxaqueca crônica e 36 com enxaqueca episódica e um grupo controle de 36 voluntários saudáveis. Os testes de equilíbrio estático e dinâmico unipodal direito/esquerdo foram realizados em cada grupo, com os olhos abertos e fechados, por meio de uma plataforma de equilíbrio posturográfico (Techno-body Prokin).

**Resultados:**

Nenhuma diferença significativa foi encontrada entre pacientes com enxaqueca episódica e crônica e indivíduos controle em relação aos valores da área de olhos abertos e olhos fechados (valores de área de olhos abertos: p=0,559, p=0,414 e p=0,906; valores de área de olhos fechados: p=0,740, p=0,241 e p=0,093, respectivamente). No entanto, os valores de área foram significativamente maiores em pacientes com enxaqueca episódica e crônica do que no grupo controle, o que indica que pacientes com enxaqueca podem ter desempenho de equilíbrio inferior. Os valores do perímetro foram relativamente mais altos, o que sustenta a hipótese de que os pacientes com enxaqueca apresentam desempenho de equilíbrio inferior. Além disso, o número médio de voltas foi significativamente menor entre os pacientes com enxaqueca do que no grupo controle, o que também implica que os pacientes com enxaqueca possam ter um desempenho de equilíbrio inferior.

**Conclusão:**

Embora nenhuma diferença significativa tenha sido detectada entre os pacientes com enxaqueca crônica e episódica e o grupo controle e entre os pacientes com enxaqueca crônica e episódica no que diz respeito ao desempenho do equilíbrio, os pacientes com enxaqueca crônica parecem ter um desempenho relativamente inferior do que os pacientes com enxaqueca episódica. Outros estudos com maior número de pacientes são necessários para investigar a relação entre esses parâmetros e o equilíbrio.

## INTRODUCTION

Migraine is defined as a neurovascular syndrome that is triggered by various factors, characterized by headache and accompanied by different symptoms^1^. Episodic migraine gradually progresses into chronic migraine, which is a more severe form. Moreover, approximately 3% of patients with episodic migraine progress into chronic migraine within a period of one year^2^^,^^3^. Chronic migraine is defined as a headache (tension-type and/or migraine headache) occurring on 15 or more days/month for more than 3 months, which has the features of migraine headache on at least 8 days/month^4^.

Besides vestibular anomalies, auras and subclinical ischemic brain lesions, balance control impairments are also highly frequent among migraine patients^5^. Although the exact mechanism of these impairments in migraine patients remains unknown, they have been attributed to subclinical cerebellar or brainstem dysfunction and to central vestibular disorders^5^^,^^6^. Nevertheless, migraine patients are considered to have normal peripheral vestibular function. It can be highlighted that balance control impairments, together with the pain associated with migraine episodes, are likely to have a negative effect on the functional abilities of patients.

Although the effects of auras and the frequency of attacks on balance have been extensively investigated in migraine patients, the effects of episodic and chronic migraine on balance have not been studied. The aim of our study was to evaluate the effects of episodic and chronic migraine on postural balance by performing static and dynamic posturographic tests, on a balance platform (Techno-body Prokin).

## METHODS

The study included 32 chronic migraine patients and 36 episodic migraine patients who presented to the neurology outpatient clinic of Van Yüzüncü Yıl University Medical School between February 2018 and April 2018 and a control group of 36 healthy volunteers. The study was started after obtaining approval from the local ethics committee. Informed consent was obtained from each participant.

The Migraine Disability Assessment Scale (MIDAS) was used to evaluate the impact of migraine headache on patients’ work, daily activities and social lives. A visual analogue scale (VAS) was used to objectively assess the severity of headache during the pain-free time.

Some migraine patients were under prophylactic treatment, including propranolol, amitriptyline, sodium valproate and topiramate.

Patients with neurological or orthopedic problems that could affect balance, or with musculoskeletal diseases, advanced hearing and vision impairment, polyneuropathy, diabetes mellitus, body mass index (BMI) of >30 or vestibular diseases, and patients that did not complete their balance tests, were excluded from the study.

Static and dynamic balance tests were performed in all three groups using a posturographic balance platform (Prokin 212-252, Pro-Kin Software Stability, TecnoBody S.r.l., Dalmine, 24044 Bergamo, Italy). All the patients were evaluated during a pain-free period. The minimum interval between pain and balance evaluation, and also between using a symptomatic drug during the pain period and this balance evaluation was stipulated as at least 48 hours.

**Figure 1:**
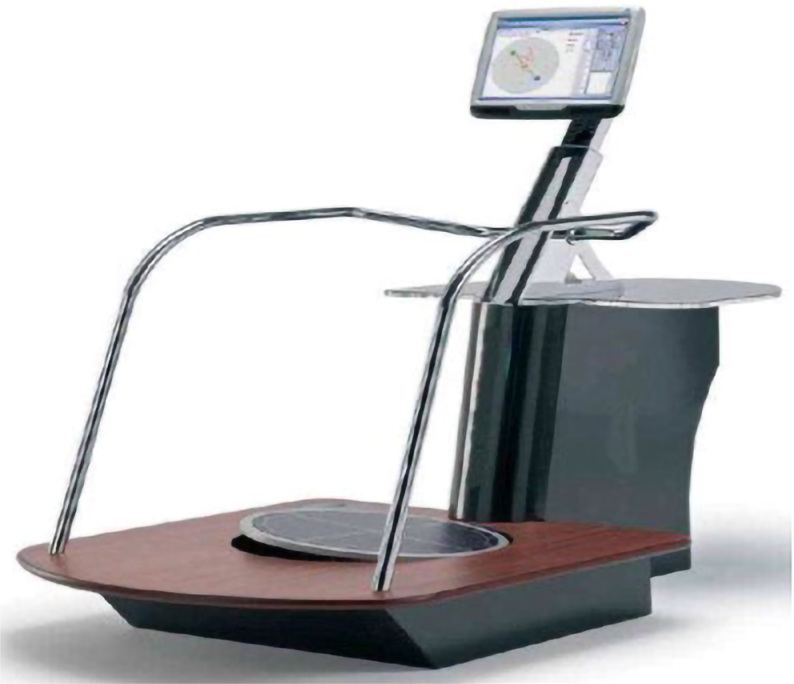
Techno-body Prokin balance measurement device.

This platform allows assessment of static balance and proprioception and can also be used for rehabilitation exercises that are performed to improve these senses. Additionally, the monitor attached to the platform provides objective live data regarding balance measurements (Figure 1).

### Static balance test

The subjects were asked to stand on the balance platform, which detected pressure sways in all directions, and were instructed to first look straight ahead at a screen surface in front of them with their eyes open for 60 sec, while trying to keep their balance on both legs, with their eyes focused on the stationary target. Subsequently, they were instructed to keep their balance on both legs with their eyes closed for another 60 sec. After these measurements, the patients were asked to try to maintain their balance for 60 sec on the right and left leg, respectively. At each interval between the tests, the subjects were allowed a resting period of 60 sec. At the end of these measurements, the device provided visual feedback regarding the length (mm) and average speed (mm/s) of body sway (total and along the anteroposterior [AP] and mediolateral [ML] axis) and the area of body sway (area of the ellipse) (mm^2^). The parameters measured in the static balance test were as follows^7^:

- Average center of pressure X (CoP-X).
- Average center of pressure Y (CoP-Y).
- Standard deviation of AP sway.
- Standard deviation of mediolateral sway.
- Average speed of anteroposterior sway (mm/s).
- Average speed of mediolateral sway (mm/s).
- Standard deviation of anteroposterior total body sway.
- Standard deviation of mediolateral total body sway.

The total length of body sway (perimeter) (mm) was calculated as the total length of the chaotic lines recorded during the patient's body sway. The shorter this length is, the better the postural balance is^8^.

Area of body sway (area of the ellipse) (mm^2^) refers to the area of a well-defined elliptical shape that covers at least 90 or 95% of the chaotic sway lines. The smaller this area is, the better the balance performance is^9^.

### Dynamic balance test

A dynamic balance test was performed using the posturographic balance platform to assess proprioception. The movable balance platform of the system works with air piston servo motors and can perform measurements in every direction with an operating angle of 15°. The subjects were asked to stand on the platform with their legs together and their hands supported on the sides. An image of three intertwined circles was then shown on the screen and the subjects were asked to rotate the cursor, which showed the net vector of the load applied on the platform by the subject, over the circle in the middle in a clockwise fashion while avoiding deviation as much as possible, at least five times within a period of 120 sec. The live feedback provided by the monitor was viewed by the subject and recorded on the device.

During the test, the average trace error (ATE) was calculated for each subject. An ATE of 0‒35% was considered very good, 35‒100% was considered adequate and >100% was considered to indicate a problem in terms of proprioceptive control. To obtain a statistically significant ATE index, the subjects needed to rotate the cursor at least five times within 120 sec^10^.

### Statistical analysis

Statistical analyses were performed using Statistical Package for the Social Sciences (SPSS) version 17.0 for Windows (Released 2008; SPSS Inc., Chicago, United States). Normal distribution of data was assessed using the Kolmogorov-Smirnov test and histogram plots. Descriptive parameters were expressed as frequencies (n), percentages (%) and mean, standard deviation (SD), median and minimum-maximum values. Variables with normal distribution (parametric data) were compared using an independent t test and variables with non-normal distribution (parametric data) were compared using the Mann-Whitney U test. Correlations were determined using Spearman's correlation coefficient. P<0.05 were considered significant.

## RESULTS

The 32 chronic migraine patients comprised 27 women (84.38%) and 5 men (15.63%), the 36 episodic migraine patients comprised 27 women (75%) and 9 men (25.00%), and the 36 healthy volunteers comprised 21 women (58.33%) and 15 men (4 1.67%). Overall, the 104 participants included 75 women (72.12%) and 29 men (27.88%). Accordingly, the female-to-male ratio was 3.8/1 among the migraine patients. The mean age was 30.53±7.02 years in the episodic group, 30.88±8.37 years in the chronic group and 28.33±6.17 years in the control group. No significant difference was found between the patient and control groups in terms of mean age.

Seven patients (four patients with chronic and three patients with episodic migraine) were excluded from the study since they could not perform the dynamic test.

No significant difference was found between the episodic and chronic groups in terms of disease duration or age at the onset of the first symptoms. The mean VAS and MIDAS scores were significantly higher in the chronic group (7.69±0.69 and 3.75±0.44, respectively) than in the episodic group (7.11±1.12 and 2.94±0.58, respectively) (p=0.019 and p<0.001).

The mean number of laps in the dynamic balance test was significantly lower in the episodic group (3.72±1.26) and chronic group (2.84±1.39) than in the control group (5.00±0.00) (p<0.001). Moreover, the mean number of laps was significantly lower in the chronic group than in the episodic group (*p=*0.008).

The mean ATE was 24.78±13.50 in the episodic group and 24.78±10.84 in the control group and no significant difference was found between the two groups (p=0.813). Moreover, the mean ATE was significantly lower in the chronic group (19.66±15.65) than in the control group (24.78±10.84) (p=0.029). In the episodic, chronic and control groups, ATE was accepted as very good in 29 (80.56%), 27 (84.38%) and 30 (83.33%) of the subjects and was accepted as adequate in 7 (19.44%), 5 (15.63%) and 6 (16.67%) of the subjects, respectively.

The eyes-open perimeter value was significantly lower in the episodic group than in the control group (541.61±149.64 vs. 605.39±117.85 mm) (p=0.048). However, the eyes-open and eyes-closed area values established that there was no significant difference between the episodic group (321.97±300.53 and 672.47±701.33 mm^2^, respectively) and the control group (265.03±131.76 and 425.94±404.95 mm^2^, respectively) (p>0.05 for both). On the other hand, these value were significantly higher in the episodic group than in the control group (Table 1).

**Table 1:** Comparison of eyes-open and eyes-closed static balance measurements between the episodic and control groups.

	EPISODIC			CONTROL			p-value
	Mean	±SD	Median	Mean	±SD	Median	
Eyes-open AP SD	4.69	±2.12	4.00	4.61	±1.71	4.00	0.725
Eyes-closed AP SD	6.11	±2.99	5.00	5.58	±2.90	5.00	0.566
Eyes-open ML SD	3.47	±1.76	3.00	3.25	±1.11	3.00	0.817
Eyes-closed ML SD	5.08	±2.73	4.00	3.78	±1.17	4.00	0.017*
Eyes-open average AP speed (mm/s)	6.06	±2.18	5.50	6.58	±1.54	7.00	0.040*
Eyes-closed average AP speed (mm/s)	8.83	±2.98	8.00	10.33	±3.76	10.00	0.056
Eyes-open average ML speed (mm/s)	4.81	±1.28	5.00	5.53	±1.38	6.00	0.021*
Eyes-closed average ML speed (mm/s)	7.33	±2.98	7.00	8.08	±3.08	8.00	0.312
Eyes-open average CoP-Y	−30.42	±26.88	−37.00	−15.81	±17.31	−17.00	<0.001*
Eyes-closed average CoP-Y	−27.78	±25.60	−31.00	−14.86	±18.64	−20.00	0.002*
Eyes-open average CoP-X	−1.56	±6.08	−1.50	−0.61	±5.49	−1.00	0.491
Eyes-closed Average CoP-X	0.39	±6.78	0.00	−0.42	±5.11	−1.00	0.571
Eyes-open trunk AP SD	5.63	±7.16	2.57	2.50	±2.11	1.66	0.149
Eyes-closed trunk AP SD	5.59	±7.04	2.41	2.45	±2.11	1.69	0.128
Eyes-open trunk ML SD	12.71	±10.69	10.19	10.09	±8.98	6.65	0.299
Eyes-closed trunk ML SD	12.83	±10.61	9.20	10.10	±9.32	6.21	0.225
Eyes-open trunk total SD	14.63	±12.01	10.41	10.76	±8.79	6.72	0.153
Eyes-closed trunk total SD	14.66	±11.92	9.75	10.79	±9.10	6.58	0.112
Eyes-open perimeter (mm)	541.61	±149.64	502.50	605.39	±117.85	614.00	0.048*
Eyes-closed perimeter (mm)	790.00	±253.86	720.00	889.25	±298.24	872.50	0.133
Eyes-open area (mm^2^)	321.97	±300.53	222.50	265.03	±131.76	253.50	0.906
Eyes-closed area (mm^2^)	672.47	±701.33	397.00	425.94	±404.95	291.00	0.093
Ratio of eyes-closed area to eyes-open area	255.75	±246.65	189.50	155.89	±83.88	133.50	0.031*
Ratio of eyes-closed perimeter to eyes-open perimeter	152.08	±47.59	136.00	146.25	±33.60	135.00	0.831

AP: anteroposterior; ML: mediolateral; SD: standard deviation; CoP: center of pressure.

* Represents statistical significance.

No significant difference was observed in terms of eyes-open and eyes-closed area values between the chronic migraine group (308.72±209.05 and 536.41±508.28 mm^2^, respectively) and the control group (265.00±131.76 and 425.94±404.95 mm^2^, respectively) (p>0.05 for both). However, these values were significantly higher in the chronic migraine group than in the control group (Table 2).

**Table 2:** Comparison of eyes-open and eyes-closed static balance measurements between the chronic and control groups.

	CHRONIC			CONTROL			p-value
	Mean	±SD	Median	Mean	±SD	Median	
Eyes-open AP SD	4.34	±1.26	4.00	4.61	±1.71	4.00	0.620
Eyes-closed AP SD	5.72	±2.49	5.00	5.58	±2.90	5.00	0.716
Eyes-open ML SD	3.59	±1.62	3.00	3.25	±1.11	3.00	0.471
Eyes-closed ML SD	4.66	±2.25	4.00	3.78	±1.17	4.00	0.086
Eyes-open average AP speed (mm/s)	6.03	±1.33	6.00	6.58	±1.54	7.00	0.151
Eyes-closed average AP speed (mm/s)	10.56	±3.21	10.00	10.33	±3.76	10.00	0.729
Eyes-open average ML speed (mm/s)	5.28	±1.30	5.00	5.53	±1.38	6.00	0.538
Eyes-closed average ML speed (mm/s)	8.47	±2.79	8.00	8.08	±3.08	8.00	0.473
Eyes-open average CoP-Y	−36.03	±18.79	−35.00	−15.81	±17.31	−17.00	<0.001*
Eyes-closed average CoP-Y	−32.22	±20.37	−33.50	−14.86	±18.64	−20.00	0.001*
Eyes-open average CoP-X	−1.06	±5.59	0.00	−0.61	±5.49	−1.00	0.738
Eyes-closed average CoP-X	−0.34	±6.79	0.00	−0.42	±5.11	−1.00	0.960
Eyes-open trunk AP SD	9.22	±10.50	4.14	2.50	±2.11	1.66	0.001*
Eyes-closed trunk AP SD	10.31	±13.29	4.64	2.45	±2.11	1.69	<0.001*
Eyes-open trunk ML SD	19.22	±10.36	18.11	10.09	±8.98	6.65	<0.001*
Eyes-closed trunk ML SD	19.37	±10.41	19.03	10.10	±9.32	6.21	<0.001*
Eyes-open trunk total SD	22.64	±12.54	25.63	10.76	±8.79	6.72	<0.001*
Eyes-closed trunk total SD	22.91	±12.70	26.12	10.79	±9.10	6.58	<0.001*
Eyes-open perimeter (mm)	567.12	±114.13	567.00	605.39	±117.85	614.00	0.180
Eyes-closed perimeter (mm)	901.44	±306.44	854.50	889.25	±298.24	872.50	0.869
Eyes-open area (mm^2^)	308.72	±209.05	272.00	265.03	±131.76	253.50	0.414
Eyes-closed area (mm^2^)	536.41	±508.28	371.00	425.94	±404.95	291.00	0.241
Ratio of eyes-closed area to eyes-open area	176.75	±95.37	165.00	155.89	±83.88	133.50	0.280
Ratio of eyes-closed perimeter to eyes-open perimeter	166.16	±45.52	159.00	146.25	±33.60	135.00	0.058

AP: anteroposterior; ML: mediolateral; SD: standard deviation; CoP: center of pressure.

* Represents statistical significance.

No significant difference was found in terms of eyes-closed perimeter values between the chronic and episodic migraine groups (901.44±306.44 and 790.00±253.86, respectively) (p>0.05). However, these values were significantly higher in the chronic migraine group than in the episodic migraine group.

No significant difference was found between the episodic and control groups with regard to right-leg perimeter (1800.56±535.20 and 1739.50±402.27 mm, respectively), left-leg perimeter (1854.28±665.12 and 1755.81±486.49 mm, respectively) and left-leg area (889.75±647.27 and 807.33±370.06 mm^2^, respectively). However, these values were found to be relatively higher in the episodic group than in the control group (Table 3).

**Table 3:** Comparison of right and left-leg static balance measurements between the episodic and control groups.

	EPISODIC			CONTROL			p-value
	Mean	±SD	Median	Mean	±SD	Median	
Right-leg average CoP-X	5.19	±7.55	3.50	4.69	±5.77	6.00	0.753
Left-leg average CoP-X	−10.92	±16.29	−7.50	−7.17	±5.93	−7.00	0.580
Right-leg average CoP-Y	−18.47	±23.66	−17.50	−7.94	±22.79	−10.00	0.059
Left-leg average CoP-Y	−25.50	±27.47	−32.50	−24.67	±18.87	−24.50	0.881
Right-leg AP SD	8.06	±2.63	8.00	8.42	±2.29	8.00	0.562
Left-leg AP SD	8.94	±3.63	8.00	8.89	±2.45	8.00	0.554
Right-leg ML SD	4.75	±1.20	4.50	4.64	±0.87	5.00	0.838
Left-leg ML SD	5.06	±1.37	5.00	4.78	±1.35	5.00	0.377
Right-leg average AP speed (mm/s)	18.78	±5.99	19.00	18.97	±4.83	17.50	0.879
Left-leg average AP speed (mm/s)	19.42	±7.35	18.50	19.22	±5.95	18.00	0.991
Right-leg average medium-lateral speed (mm/s)	19.39	±6.32	17.50	18.03	±4.33	18.00	0.433
Left-leg average ML speed (mm/s)	19.33	±7.66	18.00	18.22	±5.02	18.00	0.852
Right-leg trunk AP SD	6.98	±7.33	4.06	3.70	±2.89	2.62	0.066
Left-leg trunk AP SD	6.58	±6.51	3.88	3.29	±1.66	3.09	0.102
Right-leg trunk ML SD	12.32	±9.92	8.77	8.49	±7.49	5.02	0.056
Left-leg trunk ML SD	13.03	±10.09	9.47	9.05	±8.57	5.37	0.034*
Right-leg trunk total SD	14.80	±11.53	10.84	9.84	±7.29	7.09	0.051
Left-leg trunk total SD	14.79	±10.95	11.19	10.34	±7.87	6.85	0.035*
Right-leg perimeter (mm)	1800.56	±535.20	1771.50	1739.50	±402.27	1691.50	0.554
Left-leg perimeter (mm)	1854.28	±665.12	1854.00	1755.81	±486.49	1698.50	0.558
Right-leg area (mm^2^)	725.44	±333.78	711.00	729.22	±261.70	688.00	0.857
Left-leg area (mm^2^)	889.75	±647.27	782.50	807.33	±370.06	720.00	0.910

AP: anteroposterior; ML: mediolateral; SD: standard deviation; CoP: center of pressure.

* Represents statistical significance.

No significant difference was observed between the chronic and control groups with regard to right-leg perimeter (1830.78±596.59 and 1739.50±402.27 mm, respectively) and right-leg area (855.59±477.59 and 729.22±261.70 mm^2^, respectively). However, these values were significantly higher in the chronic group than in the control group (Table 4).

**Table 4:** Comparison of right and left-leg static balance measurements between the chronic and control groups.

	CHRONIC			CONTROL			p-value
	Mean	±SD	Median	Mean	±SD	Median	
Right-leg average CoP-X	8.12	±7.34	9.50	4.69	±5.77	6.00	0.035*
Left-leg average CoP-X	−5.50	±7.30	−3.50	−7.17	±5.93	−7.00	0.155
Right-leg average CoP-Y	−11.91	±25.06	−9.00	−7.94	±22.79	−10.00	0.497
Left-leg average CoP-Y	−19.06	±22.00	−21.50	−24.67	±18.87	−24.50	0.262
Right-leg AP SD	8.84	±3.25	8.00	8.42	±2.29	8.00	0.901
Left-leg AP SD	8.03	±2.09	8.00	8.89	±2.45	8.00	0.268
Right-leg ML SD	4.97	±1.43	5.00	4.64	±.87	5.00	0.588
Left-leg ML SD	5.03	±1.06	5.00	4.78	±1.35	5.00	0.201
Right-leg average AP speed (mm/s)	19.84	±7.15	18.50	18.97	±4.83	17.50	0.887
Left-leg average AP speed (mm/s)	19.81	±5.88	18.00	19.22	±5.95	18.00	0.749
Right-leg average medium-lateral speed (mm/s)	18.97	±6.06	18.00	18.03	±4.33	18.00	0.810
Left-leg average ML speed (mm/s)	18.94	±5.05	18.00	18.22	±5.02	18.00	0.584
Right-leg trunk AP SD	9.61	±10.16	5.76	3.70	±2.89	2.62	0.007*
Left-leg trunk AP SD	9.10	±9.11	5.28	3.29	±1.66	3.09	0.001*
Right-leg trunk ML SD	15.82	±11.00	15.07	8.49	±7.49	5.02	0.003*
Left-leg trunk ML SD	16.42	±11.26	16.05	9.05	±8.57	5.37	0.008*
Right-leg trunk total SD	19.91	±13.19	16.34	9.84	±7.29	7.09	0.001*
Left-leg trunk total SD	19.79	±12.89	18.42	10.34	±7.87	6.85	0.002*
Right-leg perimeter (mm)	1830.78	±596.59	1764.00	1739.50	±402.27	1691.50	0.749
Left-leg perimeter (mm)	1768.81	±582.60	1627.00	1755.81	±486.49	1698.50	0.868
Right-leg area (mm^2^)	855.59	±477.59	748.50	729.22	±261.70	688.00	0.597
Left-leg area (mm^2^)	790.69	±316.07	749.50	807.33	±370.06	720.00	0.975

AP: anteroposterior; ML: mediolateral; SD: standard deviation; CoP: center of pressure.

* Represents statistical significance.

No significant difference was observed between the episodic and chronic migraine groups with regard to right and left-leg static balance measurements.

No significant correlation was detected between eyes-open and eyes-closed static balance measurements and VAS, MIDAS and disease duration, whereas a moderate negative correlation was found between MIDAS and left-leg perimeter (p=0.022; r: −0.381), whereby the left-leg perimeter value decreased as the MIDAS score increased. Similarly, MIDAS also established a moderate negative correlation with eyes-open perimeter (p=0.022; r: −0.403) and right-leg perimeter (p=0.043; r: −0.360), whereby the eyes-closed and right-leg perimeters decreased as the MIDAS score increased.

## DISCUSSION

Migraine patients often present with balance control disorders, besides vestibular anomalies, auras and subclinical ischemic-like lesions^11^^,^^12^^,^^13^. The exact mechanism of balance disorders in migraine patients remains unknown. Moreover, although balance disorders have been associated with subclinical cerebellar or brainstem dysfunction^11^^,^^14^ and central vestibular dysfunction^11^^,^^15^^,^^16^, migraine patients are considered to have a normal peripheral vestibular system^6^. On the other hand, central vestibular dysfunction may be associated with ischemia of the labyrinth caused by vasospasm^17^. The CAMERA study (cerebral abnormalities in migraine, an epidemiological risk analysis) reported that silent posterior circulation infarcts increased the prevalence of hyperintense ischemic lesions in the brain stem and cerebellum in migraine patients, compared with control subjects^12^. We emphasize that, in our view, coexistence of balance control disorders and the pain associated with migraine episodes can have an adverse effect on the functional abilities of patients.

In our study, the average number of laps in the dynamic balance test was lower among migraine patients than among control subjects, which suggests that migraine patients may have lower balance performance. Additionally, the average number of laps among chronic migraine patients was lower than that of episodic migraine patients, which implies that chronic migraine patients may have lower balance performance than episodic migraine patients. On the other hand, no significant difference was found between the patient groups and the control group, which does not support the balance disorder hypothesis for migraine patients. To our knowledge, our study is the first of its kind in the literature to investigate ATE in migraine patients.

No significant differences were found among our groups with regard to eyes-open and eyes-closed area values. However, the area values were relatively higher in the episodic and chronic migraine group than in the control group, which suggests that migraine patients may have lower balance performance. Accordingly, we consider that larger number of subjects are needed in order to obtain significant results for these parameters in static balance tests.

Carvalho et al.^5^ and Carvalho et al.^18^ found significant differences between eyes-open and eyes-closed static balance measurements conducted on a planar surface and those performed on a foam surface, with regard to total area (cm^2^). These authors noted that the total area values measured on the foam surface were found to be higher among migraine patients than among control subjects and were higher among chronic migraine patients with aura than among migraine patients without aura. In our study, the eyes-open and eyes-closed area values were significantly higher among migraine patients than in the control group, while no significant difference was found between patients with chronic and episodic migraine, with regard to total area.

Ishizaki et al.^15^ found no significant difference between patients with episodic tension-type headache and control subjects, with regard to total distance (cm) of displacement of CoP measured in static balance tests with eyes open and eyes closed. However, the total distance of displacement with eyes closed was longer among migraine patients than in the control group and the balance performances of the migraine patients were worse than those of the control group. In our study, although no significant difference was found between chronic and episodic migraine patients with regard to eyes-closed perimeter values, the relatively higher perimeter values in the chronic group, compared with the episodic group, suggests that chronic migraine patients might have exhibited lower balance performance than the episodic migraine patients.

Carvalho et al. reported that total area values (cm^2^) measured in static balance tests using the right and left legs were both significantly higher among migraine patients with aura than among migraine patients without aura^5^. In our study, however, no significant difference was found between the migraine patients and the control group with regard to eyes-open right and left-leg area values, while the left-leg area values were significantly higher among episodic migraine patients than in the control group.

In our study, no significant correlation was found between VAS scores and eyes-open and eyes-closed right and left-leg static balance measurements, among both the episodic and the chronic migraine. Nevertheless, despite the absence of any significant correlation, our study is of high value since, to our knowledge, no studies in the literature have investigated the relationship between VAS scores and static balance measurements among migraine patients.

Among our patients, MIDAS scores established a moderate negative correlation with left-leg perimeter values in the episodic migraine group and established a moderate negative correlation with right-leg perimeter values measured with eyes closed in the chronic migraine group. In a similar way, our study is of high value since, to our knowledge, no studies in the literature have investigated the relationship between MIDAS scores and static balance measurements in migraine patients.

Carvalho et al. reported that the migraine patients had lower balance performance than the control group and that the presence of aura and frequent migraine attacks had an adverse effect on postural performance. These authors also noted that patients with chronic migraine and aura exhibited lower balance performance, compared with control subjects and migraine patients without aura^18^. Similarly, in our study, the eyes-closed right-leg perimeter values were significantly higher among the chronic migraine patients than among the episodic migraine patients and in the control group.

Akdal et al. evaluated 25 migraine patients without basilar migraine and vertigo (including 10 migraine patients with visual aura) and 25 control subjects and found significant deterioration in balance parameters among migraine patients, compared with the control group^14^. The same authors conducted a follow-up study with the same sample in the following year and reported that the balance disorders had persisted among the migraine patients and that some of the balance parameters had even deteriorated noticeably^16^.

Our study was limited since it had a small patient population, the groups were not sufficiently homogeneous, some patients were using prophylactic drugs that could have affected their balance performance and the balance tests were performed during a pain-free period rather than during a pain attack.

In conclusion, in the literature it is indicated that balance performance is typically lower among migraine patients than among control subjects, and reviews of the literature have indicated that no studies comparing balance performances between episodic and chronic migraine patients had been conducted. In the present study, we made this comparison of balance performances between episodic and chronic migraine patients. Although no significant difference was found between chronic and episodic migraine patients and control subjects, chronic migraine patients seemed to have lower balance performance than episodic migraine patients. On the other hand, we also found that some of the balance parameters examined in our study had not addressed in previous studies (i.e. ATE, VAS and MIDAS). Further studies with larger numbers of patients are needed, in order to investigate the relationship between these parameters and balance. We believe that our results will shed light that future studies can build on.
